# Understanding the Microenvironment of Intervertebral Disc Degeneration: A Comprehensive Review of Pathophysiological Insights and Therapeutic Implications

**DOI:** 10.3390/ijms26209938

**Published:** 2025-10-13

**Authors:** Zuzanna Ząbek, Aleksandra Wyczałkowska-Tomasik, Kamil Poboży, Jakub Piotr Adamus, Grzegorz Turek, Mirosław Ząbek, Leszek Pączek

**Affiliations:** 1 Clinical Immunology Student Scientific Association, Medical University of Warsaw, 02-006 Warsaw, Poland; zuzanna.zabek2@gmail.com (Z.Z.); jadamus.md@gmail.com (J.P.A.); 2 Department of Clinical Immunology, Medical University of Warsaw, 02-006 Warsaw, Poland; aleksandra.wyczalkowska-tomasik@wum.edu.pl (A.W.-T.); leszek.paczek@wum.edu.pl (L.P.); 3 Department of Neurosurgery, Brodnowski Masovian Hospital, 03-242 Warsaw, Poland; pobozykamil@gmail.com (K.P.); turek.grz@gmail.com (G.T.); 4 Gamma Knife Center, 03-242 Warsaw, Poland; 5 Department of Neurosurgery, Centre of Postgraduate Medical Education, 03-242 Warsaw, Poland; 6 Institute of Biochemistry and Biophysics, Polish Academy of Sciences, 02-106 Warsaw, Poland

**Keywords:** intervertebral disc degeneration, microenvironment, extracellular matrix, cytokine, collagen, inflammation, ferroptosis, oxidative stress, gene therapy, nanomedicine

## Abstract

Intervertebral disc degeneration is a leading contributor to chronic back pain and disability worldwide. This review comprehensively explores the complex interplay of cellular, molecular, and biomechanical alterations within the disc microenvironment that underlie intervertebral disc degeneration pathophysiology. Emphasis is placed on extracellular matrix degradation, cellular senescence, inflammation, oxidative stress, angiogenesis, and multiple forms of programmed cell death including apoptosis, pyroptosis, and ferroptosis. An in-depth analysis of key signaling pathways and regulatory molecules illustrates how these processes disrupt homeostasis and drive disease progression. Additionally, the review highlights emerging therapeutic approaches aimed at modifying the disc microenvironment, including mesenchymal and notochordal cell-based therapies, senolytics, ferroptosis inhibitors, gene therapy, and biomaterial innovations such as hydrogels, scaffolds, and nanocarriers. These strategies target degenerative cascades at the molecular level and represent a shift toward regenerative and disease-modifying interventions. While several approaches show promise in preclinical and early clinical studies, challenges related to safety, delivery, and long-term efficacy remain. This review underscores the importance of integrating molecular insights with translational innovations to develop targeted therapies for intervertebral disc degeneration and guide future research efforts.

## 1. Introduction

Intervertebral disc degeneration (IVDD) is a leading cause of chronic low back pain and disability worldwide [1,2,3,4]. The intervertebral disc (IVD) is a complex structure composed of the nucleus pulposus (NP), annulus fibrosus (AF), and two cartilage endplates (CEPs), which function together to provide flexibility and shock absorption to the spine [5,6,7]. With aging and pathological conditions, the disc undergoes degenerative changes that disrupt the IVD microenvironment, contributing to pain and disability [2,8]. Despite the significant burden of IVDD on healthcare systems and patient quality of life, the precise mechanisms driving its progression remain incompletely understood.

Recent advances in molecular biology, biomechanics, and regenerative medicine have provided insights into the complex interplay of biochemical, cellular, and mechanical factors within the degenerating disc microenvironment. Emerging research highlights the role of extracellular matrix (ECM) breakdown, cellular senescence, inflammation, oxidative stress, aberrant angiogenesis, immune cell infiltration, and mechanical instability in accelerating disc degeneration [1,2,3,4,5,6,7,8,9]. Understanding these pathological processes is critical for developing targeted therapies, including biological agents, cell-based approaches, and tissue engineering strategies, as current interventions mainly provide symptomatic relief without modifying the underlying disease progression [2].

Despite extensive research on the pathophysiology of IVDD, there remains a lack of comprehensive reviews that integrate the intervertebral disc microenvironment with the translational potential of recent molecular and therapeutic advances. Existing reviews have predominantly focused on specific aspects of the disease, such as inflammation, cellular senescence, or regenerative strategies, often addressing these elements in isolation. In contrast, the present review provides a holistic and integrative perspective, combining current knowledge of cellular, biochemical, and biomechanical alterations with emerging therapeutic modalities, including cell-based therapies, senolytics, ferroptosis inhibitors, gene therapy, and advanced biomaterials. By bridging fundamental mechanistic insights with translational approaches, this review aims to delineate key pathological processes, highlight novel therapeutic targets, and inform future research directions toward the development of effective disease-modifying interventions for IVDD.

## 2. Methodological Approach of the Narrative Review

This review is based on a narrative synthesis of the current literature on intervertebral disc degeneration. Relevant articles were identified through searches of PubMed, Scopus, and Web of Science databases up to September 2025, using following keywords and combinations “intervertebral disc degeneration”, “microenvironment”, “extracellular matrix”, “inflammation”, “oxidative stress”, “cell death”, “autophagy”, “pathogenesis”, “senescence”, “angiogenesis”, “nerve”, “treatment”, and “therapy”. The search focused primarily on English-language publications from the past two decades, complemented by seminal earlier studies. Both original research articles and review papers were considered. Selection was based on the authors’ assessment of the scientific relevance, novelty, and contribution of each study to the understanding of IVDD mechanisms or therapeutic strategies. The reference lists of the included papers were also screened to identify the additional relevant studies. Conference abstracts and articles not directly related to the IVDD microenvironment, pathophysiology, and treatment were excluded. The review was conducted as a comprehensive, narrative synthesis of the most relevant and influential literature in the field.

## 3. Microenvironment of a Healthy Intervertebral Disc

The intervertebral disc is a highly specialized structure that provides mechanical support, flexibility, and shock absorption within the spinal column [10]. Its microenvironment is a finely regulated system that ensures structural integrity and metabolic homeostasis. Disruptions in this microenvironment contribute to intervertebral disc degeneration, underscoring the importance of understanding its physiological state [11].

The three main components of the disc—the nucleus pulposus, annulus fibrosus, and cartilage endplate—function in a coordinated manner to maintain biomechanical and metabolic equilibrium. The NP maintains hydration and provides resistance to compressive forces, the AF ensures tensile strength and structural integrity, and the CEP facilitates nutrient exchange while preventing vascular infiltration [10,11,12]. This equilibrium is essential for disc longevity.

### 3.1. Nucleus Pulposus

The NP constitutes the central, gelatinous core of the disc and is primarily responsible for resisting compressive forces.

Mechanically, the NP functions as a hydrogel-like structure that distributes compressive loads evenly across the disc. The high water content (66–86%) provides hydrostatic pressure, which is essential for resisting spinal compression [13]. During mechanical loading, water is expelled from the NP, and upon unloading, it is reabsorbed, ensuring the preservation of disc height and function [14,15].

The extracellular matrix of the NP is rich in proteoglycans, particularly aggrecan, which binds water molecules, maintaining disc hydration and osmotic pressure. Type II collagen forms a flexible scaffold that supports the proteoglycan-rich matrix, while smaller amounts of type VI, IX and XI collagen contribute to fibril organization and stability [13].

The cellular composition of the NP changes with age, with notochordal cells (NCs) predominating in early life and chondrocyte-like NP cells becoming more prominent in adulthood [16]. These cells function in a hypoxic environment, relying predominantly on anaerobic metabolism for energy production. The biochemical environment of the NP is characterized by a balance between anabolic and catabolic processes. Growth factors, including transforming growth factor-beta (TGF-β), insulin-like growth factor-1 (IGF-1), and bone morphogenetic protein-7 (BMP-7), stimulate ECM synthesis and promote NP cell survival [17,18,19,20]. Hypoxia-inducible factor-1 alpha (HIF-1α) plays a critical role in cellular adaptation to the low-oxygen environment by upregulating genes involved in anaerobic metabolism and proteoglycan synthesis [16].

### 3.2. Annulus Fibrosus

The AF surrounds the NP and consists of concentric lamellae of collagen fibers, providing structural integrity and resistance to multidirectional mechanical forces. The outer AF is primarily composed of fibroblast-like cells that synthesize type I collagen, ensuring high tensile strength, while the inner AF contains chondrocyte-like cells that produce a mix of type I and type II collagen, facilitating the transition toward the NP [21,22]. The ECM of the AF contains moderate levels of proteoglycans, which contribute to hydration and flexibility while maintaining a fibrous structure [23].

The biomechanical properties of the AF are governed by its unique lamellar organization, in which collagen fibers are aligned in alternating oblique directions [24]. This arrangement allows the AF to withstand torsional, shear, and compressive forces while maintaining disc stability. The presence of elastin fibers further enhances the AF’s ability to restore shape following deformation.

Biochemically, both NP and AF exhibit tightly regulated ECM turnover, mediated by a controlled balance between matrix metalloproteinases (MMPs) and their inhibitors, tissue inhibitors of metalloproteinases (TIMPs) [25,26]. Mechanotransduction pathways, including Yes-associated protein (YAP)/transcriptional co-activator with PDZ-binding motif (TAZ) and integrin-focal adhesion kinase (FAK) signaling, regulate cellular responses to mechanical stimuli, ensuring appropriate ECM remodeling in response to physiological loading [27,28].

### 3.3. Cartilage Endplate

The CEP is a thin layer of hyaline cartilage that separates the IVD from the adjacent vertebral bodies. It plays a crucial role in regulating nutrient transport, providing mechanical support, and preventing vascular invasion into the avascular NP [13,29]. The CEP is composed of chondrocytes that produce type II collagen and proteoglycans, forming a semi-permeable matrix that allows for the diffusion of essential nutrients and metabolites [13]. The bony region of the CEP contains type I collagen, providing structural stability and anchoring the disc to the vertebral body [25,29].

Nutrient transport through the CEP is critical for disc homeostasis, as the IVD lacks direct vascularization (except for limited vascular penetration into the outer annulus fibrosus). Glucose and oxygen diffuse from vertebral capillaries into the disc, supporting NP and AF cell metabolism. Similarly, lactate and other metabolic byproducts are removed via passive diffusion, preventing acidification of the microenvironment [30]. The CEP also expresses anti-angiogenic factors, such as chondromodulin-I, which inhibit neovascularization and maintain the avascular nature of the disc, thereby preventing inflammatory cell infiltration and tissue fibrosis [31].

Mechanically, the CEP serves as a load-transmitting structure, distributing compressive forces between the IVD and the vertebral bodies. Its elasticity allows for subtle deformation during spinal motion, reducing stress on the NP and AF [32]. The permeability of the CEP is essential for maintaining disc hydration and nutrient exchange, ensuring the longevity of disc function [29,30,31,32].

## 4. Cellular and Molecular Mechanisms Underlying Intervertebral Disc Degeneration

**Figure 1:**
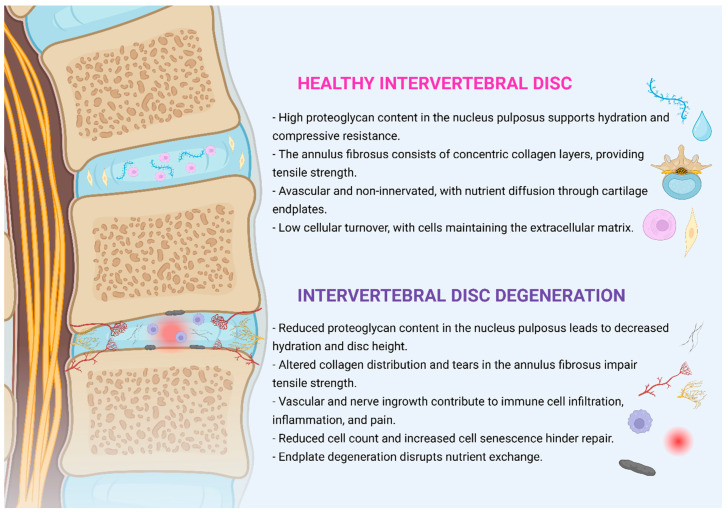
**Structural and Biochemical Comparison Between a Healthy Intervertebral Disc and a Degenerated Disc.** In the healthy intervertebral disc, high proteoglycan content within the nucleus pulposus ensures effective hydration and resistance to compressive loads. The annulus fibrosus maintains tensile integrity through its organized, concentric collagen lamellae. These discs are avascular and non-innervated, relying on diffusion through intact cartilage endplates for nutrient supply. Cell turnover remains low, with resident cells actively maintaining extracellular matrix homeostasis. In contrast, the degenerated disc shows marked proteoglycan depletion, resulting in diminished hydration and loss of disc height. Structural integrity is compromised by disorganized and fragmented collagen in the annulus fibrosus. Pathological ingrowth of blood vessels and nerves facilitates immune cell infiltration and chronic inflammation, contributing to discogenic pain. Increased cellular senescence and reduced cell density impair repair mechanisms, while endplate degeneration disrupts nutrient transport, further exacerbating degeneration. Created in BioRender. https://BioRender.com/h6q9z72 (URL accessed on 6 October 2025).

Disruption of the intricate interplay among the nucleus pulposus, annulus fibrosus, and cartilage endplates initiates a cascade of cellular and molecular events that drive intervertebral disc degeneration. These processes manifest structurally through proteoglycan loss, collagen disorganization, and endplate damage, and biochemically through inflammation, oxidative stress, and cellular senescence. Together, these alterations impair the disc’s ability to maintain its structural integrity and biomechanical function. Figure 1 provides a visual summary of the key structural and biochemical differences between healthy and degenerated discs, illustrating how these changes converge to promote disease progression.

### 4.1. Phenotypic Shift in Nucleus Pulposus Cells

Notochordal cells are the embryonic progenitors of NP cells and play a crucial role in maintaining disc homeostasis during early life. NCs are largely lost with age, giving way to smaller, rounded chondrocyte-like NP cells (derivatives of notochordal lineage) with reduced anabolic, anti-inflammatory and anti-apoptotic properties [16,33,34,35]. With disc degeneration, the NP tissue becomes fibrotic and dehydrated as proteoglycan content declines, and the normally gelatinous nucleus is replaced by fibrocartilaginous matrix. NP cells in degenerated discs often proliferate in situ, forming cell clusters especially in areas of matrix fissures [22,36,37]. These cell clusters are a histological hallmark of degeneration and are thought to be an attempted repair response. The overall cell density decreases due to various types of cellular death [38].

Surviving NP cells adopt a catabolic and pro-inflammatory phenotype, characterized by increased expression of cytokines such as Interleukin-1 beta (IL-1β) and tumor necrosis factor-alpha (TNF-α), upregulation of matrix-degrading enzymes including MMPs and ADAMTS (a disintegrin and metalloproteinase with thrombospondin motifs) aggrecanases, and a concomitant reduction in the synthesis of key extracellular matrix components such as aggrecan and type II collagen [22].

One of the most significant cellular changes in IVDD is the emergence of fibroblast-like cell populations in the NP, contributing to fibrotic remodeling [39]. These cells originate from resident NP cells via fibroblastic/myofibroblastic transition (induced by TGF-β), and from circulating myeloid-derived fibrocytes. This fibrotic transformation is associated with a shift from type II to type I collagen production, and with the upregulation of fibronectin and small leucine-rich proteoglycans like biglycan, ultimately altering the mechanical properties of the disc [39,40]. Fibroblastic NP cells were shown to upregulate matrix-remodeling genes such as HTRA1 (High-temperature requirement protein A1) and ANGPTL4 (Angiopoietin-like 4), markers associated with severe disc degeneration [40].

### 4.2. Phenotypic Alterations of Annulus Fibrosus Cells

During degeneration, the AF undergoes structural disorganization and the cell phenotype shifts accordingly. Altered loading patterns, resulting from aging, injury, or progressive disc collapse, exert abnormal tensile and shear forces on the lamellar structure. These changes compromise the highly ordered collagen architecture, leading to splitting, interweaving, and the formation of radial and circumferential tears [38,41,42]. In early stages of degeneration, small clusters of proliferating AF cells can emerge within the tears, resembling the clustering observed in the nucleus pulposus; in severely degenerated or herniated discs, these clusters become larger and more prominent. While these proliferative foci likely represent an attempt at tissue repair, the overall cell number in the AF decreases, reflecting a net loss of cells similar to that seen in the NP [43]. The resident AF cells in a degenerated disc also change character. They can adopt a more fibrotic or hypertrophic phenotype, increasing production of collagens typically seen in scar tissue (e.g., type III collagen) and even expressing markers more characteristic of tendon/ligament cells. For example, degenerated human AF cells show up-regulation of tenomodulin, a tendon-related proteoglycan, compared to normal AF cells [22]. These changes are influenced by altered TGF-β signaling, which initially promotes matrix deposition but, under chronic stimulation, can drive fibrosis and abnormal tissue remodeling. Concurrently, exposure to sustained mechanical stress and microdamage triggers the release of pro-inflammatory cytokines (e.g., interleukins, TNFs) and matrix metalloproteinases, amplifying matrix degradation and perpetuating a feed-forward loop of catabolism [22,44]. Overall, the degenerated AF is characterized by loss of the normal outer-vs-inner cell distinction (as the structure breaks down), emergence of cell clusters, and cell death.

### 4.3. Structural and Cellular Changes in the Cartilage Endplate

With age and degeneration, the CEP undergoes progressive structural and cellular changes that profoundly impact disc homeostasis. One key alteration is pathological calcification of the CEP [45]. This process is not merely passive mineral deposition; it involves active remodeling of the matrix and dysregulation of local signaling pathways, including upregulation of osteogenic and hypertrophic markers in resident chondrocytes. Calcification increases the CEP’s stiffness and decreases its permeability, impairing the bidirectional transport of nutrients and metabolic waste between the vertebral capillaries and the disc interior [46,47]. As a result, cells in both the NP and AF are deprived of glucose and oxygen, creating a hypoxic, acidic microenvironment that accelerates catabolic and apoptotic responses.

The degenerated CEPs often appear thinner and more porous than normal, and they may develop cracks or fissures [48,49]. The healthy CEP’s chondrocyte population is progressively lost or phenotypically altered in degeneration—cartilage cells either die or undergo hypertrophy and terminal differentiation, expressing markers such as type X collagen, alkaline phosphatase, especially in areas undergoing calcification [50,51].

### 4.4. Biomechanical Causes of Intervertebral Disc Degeneration

Mechanical loading constitutes a central determinant in the initiation and progression of intervertebral disc degeneration. Beyond age-related changes, abnormal mechanical conditions, including excessive compression, torsion, tension, spinal instability, and misalignment, act as potent accelerators of degenerative cascades [1,4]. Localized mechanical insults, such as endplate fractures or annular delamination, disrupt the structural and functional interplay among the NP, AF, and CEPs, thereby promoting structural failure and predisposing the disc to herniation [4].

Importantly, disc cell metabolism is directly regulated by mechanical cues: physiological hydrostatic pressure stimulates proteoglycan synthesis, whereas static, excessive, or insufficient loading suppresses anabolic activity and enhances catabolic enzyme production, followed by a transition from type II to type I collagen within the NP. These changes ultimately lead to increased tissue stiffness and disc height reduction, promoting further structural damage [10].

Repetitive injurious loading leads to cumulative microstructural damage within the AF, resulting in annular tears and herniation. Structural alterations in adjacent vertebrae, facet joints, and ligaments further exacerbate these changes through both biomechanical and inflammatory mechanisms [4,9].

Compromised nutrient diffusion due to endplate calcification amplifies the deleterious effects of abnormal loading by impairing matrix synthesis and cell viability [9].

Epidemiological and experimental evidence consistently demonstrates that occupational overload, obesity, smoking, and injury contribute to pathological loading patterns, increasing both the risk and severity of disc degeneration [1,38].

Collectively, these data establish mechanical factors as the important drivers and amplifiers of the molecular and cellular mechanisms underlying IVDD pathogenesis.

### 4.5. Cellular Senescence

Cellular senescence is an evolutionarily conserved mechanism characterized by an irreversible arrest of cell proliferation in response to a variety of intrinsic and extrinsic stressors. In the IVD, senescence plays a pivotal role in aging and degeneration by reducing the pool of functional cells and altering the tissue microenvironment [52,53].

Senescence in disc cells can be broadly categorized into two main types: replicative senescence, which arises from telomere attrition, and stress-induced premature senescence (SIPS), which is triggered by genotoxic insults, oxidative stress, inflammatory cytokines, and aberrant mechanical loading [52]. In both cases, the senescence response is mediated by canonical molecular pathways involving the activation of cyclin-dependent kinase inhibitors such as p16^INK4a^ and p21^CIP1^, as well as tumor suppressor p53 and retinoblastoma protein (Rb). These pathways converge to induce G1 cell cycle arrest and block cellular proliferation [53]. Concurrently, senescent disc cells undergo morphological changes, exhibit increased senescence-associated β-galactosidase (SA-β-gal) activity, and display shortened telomeres, particularly in the degenerative NP tissue [54].

Senescent disc cells are not merely quiescent; they adopt a senescence-associated secretory phenotype (SASP), characterized by the elevated production of pro-inflammatory cytokines (e.g., IL-1β, IL-6, TNF-α), chemokines, MMPs, and ADAMTS enzymes [55]. These secreted factors propagate catabolic processes, promote inflammation, and can induce paracrine senescence in neighboring cells, thereby perpetuating tissue deterioration. SASP activity in senescent disc cells significantly contributes to extracellular matrix breakdown, reduced synthesis of aggrecan and type II collagen, and enhanced matrix degradation, ultimately accelerating the degenerative process [55,56,57,58].

### 4.6. Autophagy Impairment

Autophagy is a fundamental catabolic mechanism responsible for the degradation of cytoplasmic macromolecules and organelles via the lysosomal pathway, ensuring cellular homeostasis, survival, and energy balance [59]. Dysregulation of this process is now recognized as a contributing factor in the pathogenesis and progression of IVDD.

Due to its avascular nature, the IVD functions under conditions of chronic nutrient deprivation, hypoxia, low pH, and oxidative stress. These stressors are known to activate autophagy under physiological conditions, enabling NP cells to degrade dysfunctional organelles, misfolded proteins, and other cellular debris to maintain their viability and metabolic equilibrium [59,60].

The mTOR (mammalian target of rapamycin) signaling pathway has been identified as a central regulator of autophagy in disc cells. Under normal nutrient-rich conditions, mTORC1 (mammalian target of rapamycin complex 1) inhibits autophagy by phosphorylating ULK1 (Serine/threonine-protein kinase ULK1), thereby preventing autophagosome initiation. Conversely, nutrient deprivation or energy stress activates AMPK (AMP-activated protein kinase), which inhibits mTORC1 and activates ULK1 to promote autophagy [60,61]. In degenerative discs, the dysregulation of these pathways—often exacerbated by aging, inflammation, or oxidative injury—leads to attenuated autophagic responses characterized by intracellular accumulation of damaged mitochondria and misfolded proteins, and decreased expression of autophagy markers such as LC3-II (LC3-phosphatidylethanolamine conjugate), Beclin-1, and Atg (autophagy-related) proteins [60,62]. The suppression of autophagy correlates with increased activity of MMPs and ADAMTS, and decreased synthesis of ECM components including aggrecan and collagen II. Moreover, insufficient autophagic clearance may induce endoplasmic reticulum (ER) stress, mitochondrial dysfunction, and inflammation—cascades that further amplify tissue degeneration [62,63]. Furthermore, while moderate activation of autophagy serves a protective role, dysregulated autophagy may accelerate cell death through apoptosis or necroptosis, particularly under conditions of sustained stress [62].

### 4.7. Programmed Cell Death Pathways in IVDD

Programmed cell death is a key driver of disc degeneration, contributing to progressive cell loss, matrix breakdown, and inflammation. Various stressors within the degenerating disc (such as nutrient deprivation, altered mechanical loading, oxidative stress, and inflammatory mediators) activate distinct but interconnected death pathways. Apoptosis, pyroptosis, necroptosis, and ferroptosis may occur simultaneously or sequentially, often converging on shared molecular signals that amplify degenerative cascades.

#### 4.7.1. Apoptosis

Apoptosis, or programmed cell death, is a central mechanism contributing to the progressive cellular depletion observed in IVDD. In healthy discs, apoptosis is tightly regulated to maintain tissue homeostasis; however, in the degenerative cascade, the dysregulation of apoptosis leads to a significant reduction in viable cells, impairing ECM turnover and structural integrity [64].

The activation of apoptosis in the IVD occurs through both intrinsic (mitochondrial) and extrinsic (death receptor-mediated) pathways. The intrinsic pathway is predominantly driven by mitochondrial dysfunction and is characterized by an increased ratio of pro-apoptotic Bax to anti-apoptotic Bcl-2, leading to cytochrome c release and subsequent activation of caspase-9 and effector caspase-3. The extrinsic pathway is triggered by inflammatory mediators such as TNF-α and Fas ligand (FasL), which bind to their respective receptors and activate caspase-8. Both pathways converge on the execution phase of apoptosis, culminating in DNA fragmentation and cell shrinkage [64,65].

Inflammatory factors including TNF-α stimulate the release of exosomes containing miR-16 microRNA, which promote apoptosis by downregulating the IGF-1/IGF-1R axis. This repression leads to deactivation of the PI3K/Akt signaling pathway, a critical pro-survival cascade, thereby enhancing caspase-3 activity and promoting apoptotic progression [66]. Similarly, gremlin-1 and periostin, which are upregulated in degenerated discs, induce apoptosis in NP cells by suppressing TGF-β-mediated Smad2/3 phosphorylation, and activation of the Wnt/β-catenin signaling cascade, respectively [67,68].

Beyond mitochondrial and receptor-mediated routes, ER stress also contributes to disc cell apoptosis under conditions of nutrient deprivation, oxidative stress, and unphysiological mechanical loading [65].

#### 4.7.2. Necroptosis

Necroptosis is a regulated form of programmed cell death that morphologically resembles necrosis but is governed by a genetically encoded signaling pathway [66].

The activation of necroptosis in IVD cells has been linked to a variety of pathological triggers including pro-inflammatory cytokines (e.g., TNF-α, IL-1β), mechanical compression, oxidative stress, mitochondrial dysfunction, and ER stress. Upon stimulation by these factors, RIPK1 (Receptor-interacting serine/threonine-protein kinase 1) interacts with RIPK3 (Receptor-interacting serine/threonine-protein kinase 3) via their RIP homotypic interaction motif (RHIM) domains, forming a necrosome complex. RIPK3 subsequently phosphorylates mixed lineage kinase domain-like protein (MLKL), which undergoes oligomerization and translocates to the plasma membrane to induce membrane disruption and cell lysis [69,70,71]. The release of damage-associated molecular patterns (DAMPs) further amplifies local inflammation and matrix degradation [72].

Recent studies have illuminated multiple mechanistic pathways by which necroptosis contributes to IVDD. Interferon regulatory factor 1 (IRF1) and MyD88-dependent pathway, commonly activated by Toll-like receptors, play a pivotal role in mediating RIPK3 and MLKL activation in IVD cells [72,73].

#### 4.7.3. Pyroptosis

Pyroptosis is an inflammatory form of programmed cell death characterized by cell swelling, plasma membrane rupture, and release of pro-inflammatory cytokines such as IL-1β and interleukin-18 (IL-18). Pyroptosis is primarily mediated by the cleavage of gasdermin family proteins, particularly gasdermin D (GSDMD), by inflammatory caspases [74,75]. Inflammasome activation in IVD cells may be driven by diverse upstream stimuli including oxidative stress, mitochondrial dysfunction, and ER stress—conditions prevalent in the harsh disc microenvironment [75,76]. Pyroptosis leads to uncontrolled release of inflammatory contents, which perpetuates a cycle of cell death, inflammation, and ECM degradation. Elevated expression of NLRP3 (NLR family pyrin domain containing 3), caspase-1, and GSDMD has been observed in degenerative human disc tissues, correlating with the severity of disc pathology [76].

At the molecular level, pyroptosis is initiated via canonical or noncanonical inflammasome pathways [74,75,76,77]. In the canonical pathway, pattern recognition receptors such as NLRP3, AIM2 (absent in melanoma 2), and NLRC4 (NLR family CARD domain-containing protein 4) detect pathogen-associated molecular patterns (PAMPs) or DAMPs, leading to the assembly of inflammasomes composed of the sensor protein, ASC (apoptosis-associated speck-like protein containing a CARD), and caspase-1. Activated caspase-1 cleaves GSDMD, liberating its N-terminal fragment (GSDMD-NT), which oligomerizes and forms transmembrane pores that result in cell lysis and cytokine release. In noncanonical pyroptosis, caspase-4/5 directly sense intracellular LPS and likewise cleave GSDMD, inducing pyroptosis independently of canonical inflammasomes [77]. In addition, the role of gasdermin family members beyond GSDMD is gaining attention. Gasdermins A–E, are involved in potential crosstalk between apoptosis and necroptosis. For instance, gasdermin E (GSDME), typically cleaved by caspase-3, can convert apoptotic signals into pyroptotic outcomes, especially under stress conditions, suggesting plasticity among cell death pathways in NP cells [74].

Various pathways such as PINK1-PARK2 and PKM2-EIF2AK2 modulate inflammasome activity and gasdermin pore formation. Interestingly, the ESCRT (endosomal sorting complex required for transport) machinery, typically involved in membrane repair, was shown to modulate pyroptosis by counteracting GSDMD-induced membrane rupture, highlighting a potential regulatory checkpoint in disc cell fate [75].

#### 4.7.4. Ferroptosis

Ferroptosis is an iron-dependent form of regulated cell death characterized by the accumulation of lipid peroxides and reactive oxygen species (ROS) [78]. In the context of IVDD, an increasing body of evidence has positioned ferroptosis as a critical pathological mechanism contributing to the loss of NP cell viability, oxidative damage, and chronic inflammation [79].

Mechanistically, ferroptosis arises from a disruption of intracellular redox homeostasis. It is primarily mediated by impaired activity of glutathione peroxidase 4 (GPX4), glutathione (GSH) depletion, and system Xc^−^ (comprising SLC7A11 and SLC3A2) dysfunction, which collectively disable the cellular defense against lipid peroxidation. Concurrently, an increase in intracellular ferrous iron (Fe^2+^) fuels Fenton reactions that generate hydroxyl radicals and promote ROS accumulation, initiating peroxidation of polyunsaturated fatty acids (PUFAs) within membrane phospholipids [80,81]. These lipid peroxides compromise membrane integrity, leading to cellular collapse with characteristic morphological features, including mitochondrial shrinkage, increased membrane density, and loss of cristae, but without nuclear condensation [81]. Ferroptotic cells may release DAMPs that exacerbate inflammation and immune cell recruitment within the avascular disc space.

In degenerative disc cells, transferrin receptor (TFR1) expression is elevated, increasing iron uptake, while the iron export protein ferroportin (FPN/SLC40A1) is often suppressed. This imbalance leads to intracellular iron overload, driving ROS generation and lipid peroxidation via lipoxygenase (LOX) enzymes. Factors including ACSL4 (acyl-CoA synthetase long chain family member 4) and LPCAT3 (lysophosphatidylcholine acyltransferase 3) mediate PUFA esterification into membrane phospholipids, sensitizing cells to ferroptotic death. Furthermore, ferritinophagy, a selective autophagic process mediated by NCOA4, releases labile iron from ferritin stores, further amplifying ferroptosis risk in degenerative discs [78].

The antioxidant defense axis, centered on the system Xc^−^–GSH–GPX4 pathway, is frequently impaired in IVDD. Decreased expression of SLC7A11 limits cystine uptake, reducing GSH synthesis and consequently GPX4 activity. The loss of GPX4, a selenoenzyme essential for detoxifying lipid peroxides, is a defining feature of ferroptosis and has been consistently observed in both experimental and human IVDD tissues [82].

### 4.8. Hypoxia, Nutrient Deprivation, and Aberrant Angiogenesis

In vivo measurements and modeling have confirmed that oxygen tension within the center of the NP can fall as low as 0.3–1.06%, while higher concentrations (8–10%) are observed closer to the endplate [83]. Despite this hypoxic milieu, IVD cells, especially those in the NP, are metabolically adapted to low oxygen availability. These cells primarily utilize anaerobic glycolysis, as demonstrated by their high expression of hypoxia-inducible factors (HIF-1α and HIF-2α), which regulate genes crucial for cell survival, matrix production, and energy metabolism in the absence of oxygen [83]. Cartilaginous endplate calcification impairs the diffusion of essential nutrients such as glucose and oxygen into the disc core [46,47]. This exacerbates hypoxic and nutrient-deficient conditions, which have been implicated in progressive cell death, loss of matrix integrity, and overall disc degeneration.

While the healthy IVD remains largely avascular, degeneration is frequently accompanied by neovascularization. Pro-inflammatory cytokines such as IL-1β and TNF-α are elevated in degenerated discs and can upregulate the expression of angiogenic factors including vascular endothelial growth factor (VEGF) in NP cells [84]. VEGF is further enhanced under hypoxic conditions via the stabilization and activation of HIF-1α, initiating endothelial proliferation and vessel ingrowth. These newly formed vessels may act as conduits for immune cell infiltration, thereby intensifying inflammation and contributing to matrix breakdown [85,86].

### 4.9. Nerve Ingrowth and Pain Sensitization

In healthy intervertebral discs, innervation is limited to the outer annulus fibrosus. However, degeneration facilitates sensory and sympathetic nerve ingrowth into deeper regions, including the inner annulus and nucleus pulposus [87]. This process is mediated by increased expression of neurotrophins such as nerve growth factor (NGF) and brain-derived neurotrophic factor (BDNF), along with pain-related neuropeptides like substance P and calcitonin gene-related peptide (CGRP) [85,88]. Inflammatory cytokines including IL-1β and TNF-α upregulate these molecules, promoting nerve survival and sensitization. Notably, nerves have been detected deep within the degenerate nucleus pulposus even in the absence of vascularization, often in proximity to matrix fissures [89]. This aberrant innervation is strongly associated with discogenic pain [85].

### 4.10. Oxidative Stress and Organelle Dysfunction

Oxidative stress, characterized by an imbalance between ROS production and antioxidant defenses, is a critical driver of IVDD [90]. Mitochondrial dysfunction exacerbates ROS accumulation, leading to DNA, protein, and lipid damage, and triggering cell senescence, apoptosis, and extracellular matrix breakdown [91]. Additionally, ER stress and disrupted mitochondrial quality control intensify oxidative injury and impair cellular homeostasis [92]. A vicious cycle of ROS-induced mitochondrial damage and further ROS production accelerates degeneration. The decline in antioxidant enzymes, such as superoxide dismutase (SOD) and glutathione peroxidase, further aggravates redox imbalance [93]. Collectively, organelle stress linked to oxidative damage is central to IVDD pathology, promoting senescence and apoptosis through ROS-mediated signaling cascades.

### 4.11. Inflammatory Signaling

Inflammation constitutes a central pathological mechanism in IVDD, orchestrated by both resident disc cells and infiltrating immune cells. Among the predominant mediators are the pro-inflammatory cytokines IL-1β and TNF-α, which are markedly upregulated in degenerated discs and contribute to ECM degradation, cellular senescence, apoptosis, pyroptosis, ferroptosis, and nociceptive sensitization [94,95].

TNF-α, synthesized by disc and immune cells, exists in transmembrane (tmTNF-α) and soluble (sTNF-α) forms, the latter generated via cleavage by TNF-α–converting enzyme (TACE). It exerts its effects via Tumor necrosis factor receptor 1 and 2 (TNFR1 and TNFR2) signaling, leading to activation of the NF-κB (Nuclear factor kappa-light-chain-enhancer of activated B cells) and MAPK (Mitogen-activated protein kinase) pathways [95,96,97]. IL-1β signals through the IL-1RI/IL-1RAcP complex, engaging the MyD88/IRAK/TRAF6 axis and converging on similar downstream pathways [96]. These cascades upregulate catabolic enzymes such as MMP-1, -3, -7, -9, -10, -13 and ADAMTS-4, -5, -9, -15, promoting matrix breakdown [98,99].

Both cytokines further enhance inflammation by inducing IL-6, IL-8, IL-17, CCL2 (Chemokine ligand 2), CCL3, CCL5, prostaglandin E2 (PGE2), inducible nitric oxide synthase (iNOS), and nitric oxide (NO), thereby perpetuating immune cell recruitment and activation [98,99]. IL-1β additionally promotes mitochondrial apoptosis through Bax, caspase-3/9 activation, Bcl-2 downregulation, and drives pyroptosis via NLRP3 inflammasome-mediated caspase-1 activation and Gasdermin D cleavage. IL-1β-induced oxidative stress also precipitates ferroptosis, marked by reactive oxygen species accumulation, lipid peroxidation, and suppression of GPX4 [99,100].

Interleukin-6 (IL-6) is another key cytokine in IVDD. IL-6 stimulation of disc cells induces STAT3 (Signal transducer and activator of transcription 3) phosphorylation and increases production of cyclooxygenase-2 (COX-2) and MMP-13, both of which contribute to disc inflammation and matrix catabolism [101,102]. Additional cytokines implicated in IVDD include IL-2, IL-4, IL-10, IL-12, IL-17, and interferon-γ (IFN-γ), as well as chemokines such as CCL2 (chemokine ligand 2) and CCL5, further amplifying the degenerative and inflammatory milieu [103,104,105]. Table 1 summarizes the key cytokines and chemokines implicated in the inflammatory processes of intervertebral disc degeneration, along with their primary functions and associated cellular effects.

### 4.12. Immune Cell Infiltration

As a mostly avascular organ, the healthy intervertebral disc is an immune-privileged site isolated from the systemic immune system by the intact AF and CEPs. This “blood–disc barrier” keeps the NP sequestered from immune surveillance. NP cells even express immunomodulatory molecules like FasL that induce apoptosis of infiltrating T cells and macrophages, forming a molecular barrier against immune attack [107]. However, with degeneration, this immune privilege is compromised. Annular fissures and endplate defects allow blood vessels and immune cells to infiltrate previously isolated regions of the disc. Exposed NP tissue is recognized as “foreign” by the immune system, triggering an inflammatory reaction [107,108].

Macrophages are key innate immune cells that critically shape the inflammatory microenvironment in IVDD through their dynamic infiltration, activation, and polarization. Macrophages infiltrate the disc and polarize into pro-inflammatory (M1) and anti-inflammatory (M2) phenotypes.

The recruitment and M1 polarization of macrophages is mediated by inflammatory chemokines such as CCL2, CCL3, and CX3CL1 [109,110]. M1 macrophages secrete TNF-α, IL-1β, IL-6, IL-18, and nitric oxide, promoting ECM degradation, pyroptosis, and pain [109]. Macrophages further influence disc cell activities by modulating proliferation, senescence, and programmed cell death. Their secreted cytokines activate key pathways including NF-κB, MAPK, JAK/STAT, and NLRP3 inflammasome, establishing a feedback loop that perpetuates inflammation. Furthermore, senescent NP cells actively shape the local immune microenvironment by releasing exosomes that promote M1 macrophage polarization [106]. This interplay contributes to sustained catabolic signaling, increased matrix degradation, and progressive structural deterioration of the disc.

Conversely, M2 macrophages exert regulatory and reparative effects. Through the secretion of IL-10, VEGF, MMP-7, and MMP-8, as well as exosomes that activate the TGF-β/Smad3 pathway in NP cells, M2 macrophages promote the synthesis of key extra-cellular matrix components such as aggrecan and collagen type II, counteracting matrix breakdown and supporting tissue remodeling [106,109].

The bidirectional interactions between senescent NP cells and macrophage subtypes illustrate how immune-cellular crosstalk influences ECM metabolism and disc integrity. Notably, macrophage phenotype distribution shifts with disease stage, with M1 dominance early and M2 enrichment during later repair phases [106,110,111,112].

## 5. Extracellular Matrix Remodeling

### 5.1. Alterations of ECM Components During Disc Degeneration

Intervertebral disc degeneration is characterized by pronounced changes in the quantity, quality, and organization of ECM components. A central feature is the loss and fragmentation of aggrecan and other proteoglycans in the NP [113]. With aging and degeneration, aggrecan monomers are cleaved by proteolytic enzymes into smaller, non-aggregating fragments that often diffuse out of the disc, leading to a loss of glycosaminoglycans, disc dehydration, and reduced ability to maintain height under load [113,114,115]. Type II collagen in the NP undergoes denaturation and breakdown as well. There is also an aberrant accumulation of type I collagen in regions that normally are rich in type II (such as the NP and inner AF), reflecting a phenotype switch of matrix production [115]. Collagen cross-linking is also altered: with age, advanced glycation end-products (AGEs) accumulate on long-lived collagen fibers, promoting non-enzymatic crosslinks that stiffen the matrix and make it more prone to brittle failure. This shift from a gelatinous proteoglycan-rich matrix to a more fibrous collagenous matrix is a hallmark of degeneration [40,115,116,117,118].

The levels of another component, fibronectin, markedly increase and are often present in fragmented form. While this upregulation may represent a cellular response to injury, fibronectin fragments can exacerbate degeneration by suppressing aggrecan synthesis and enhancing matrix metalloproteinase expression in disc cell cultures [40]. Similarly, small leucine-rich proteoglycans (SLRPs) such as decorin and biglycan, typically found in low quantities, become disproportionately elevated relative to aggrecan. Elastin fibers, despite their durability, can become disrupted—damage to elastin or its integration with collagen may compromise the disc’s elastic recoil [115,119].

### 5.2. Matrix-Degrading Enzymes

Matrix degradation in IVDD is driven by a variety of proteases, with MMPs and ADAMTS family being particularly important [115]. MMPs are Zn^2+^-dependent endopeptidases capable of degrading collagens, proteoglycans, and other matrix components [120]. In healthy adult discs, MMP expression is very low, but it rises markedly in consequence of mechanical stress, inflammatory cytokine activity, oxidative stress, and cellular senescence. Analyses of human disc tissues show that MMP levels correlate positively with the severity (grade) of degeneration [115,121,122,123,124,125].

Multiple MMPs have been identified in degenerated human discs, including the interstitial collagenases MMP-1, and -13 (which cleave fibrillar collagens I and II), the gelatinases MMP-2 and -9 (which degrade denatured collagen and gelatin), and stromelysins like MMP-3 (which can digest proteoglycans and non-fibrillar collagens) [126]. Among these, MMP-3 (stromelysin-1) appears to play a key role, as it is one of the most strongly upregulated enzymes in degenerated discs and can activate other pro-MMPs. MMP-3 and MMP-13 increases have been closely linked to disc matrix catabolism, and MMP-3 expression tends to rise early in degeneration, potentially initiating collagen II and aggrecan breakdown [126,127,128,129,130]. Table 2 provides an overview of the main MMPs involved in IVDD, outlining their specific substrates and contributions to extracellular matrix breakdown.

The ADAMTS family contributes major aggrecan-degrading activity. In particular, ADAMTS-4 and ADAMTS-5 (aggrecanase-1 and -2) cleave the aggrecan core protein at specific sites, causing loss of the aggregating proteoglycan [115,131]. These aggrecanases are found at elevated levels in degenerated human discs. Inflammatory cytokines present in the degenerative milieu (like IL-1β) further upregulate ADAMTS expression; for instance, ADAMTS-5 production can be induced via NF-κB and syndecan-4 signaling in NP cells exposed to cytokines [129,132]. In vitro and in vivo models reinforce the importance of aggrecanases: rodent disc compression injuries lead to high ADAMTS-4/5 expression and precipitous loss of proteoglycans, while direct injection of ADAMTS-4 into healthy disc tissue causes rapid proteoglycan depletion, collagen loss, and cell death, recapitulating degenerative changes [131]. ADAMTS-1, -7, -9, -12, and -15, have also been found to be elevated in degenerated human discs as well. ADAMTS-7 has been linked to the degradation of cartilage oligomeric matrix protein in cartilage, and in disc it may mediate some of the catabolic effects of inflammatory stimuli. The broad upregulation of multiple MMPs and ADAMTS in IVDD underscores a pathologic proteolytic cascade that dismantles the ECM [129,132,133,134,135].

Described enzymes are normally kept in check by endogenous inhibitors. Tissue inhibitors of metalloproteinases (TIMPs) bind active MMPs (and some ADAMTS) in a 1:1 ratio, blocking their activity. In the disc, TIMP-1, -2 (and to a lesser extent TIMP-3) are expressed by resident cells. During degeneration, TIMP-1 levels often increase alongside MMPs as a compensatory response. However, this rise in TIMP expression is usually insufficient to counteract the excessive protease activity. An imbalance favoring proteases thus persists, tipping the scales toward ECM breakdown [30,130,136,137,138].

Other proteolytic enzymes also play a role in disc remodeling. Cathepsins, particularly cathepsins B, L, and D, are present in human discs and can degrade collagen and aggrecan in the acidic conditions that may occur in nutritionally compromised regions of the disc, where lactic acid accumulation lowers the tissue pH [38].

**Figure 2:**
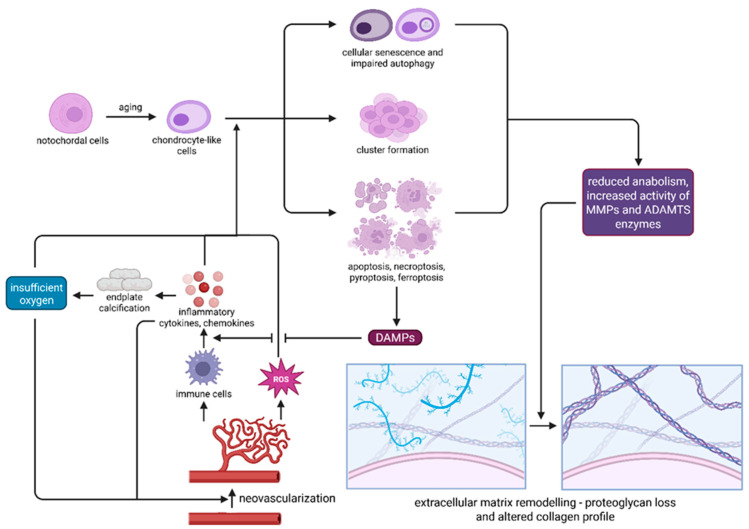
**Cellular and Molecular Mechanisms Driving Intervertebral Disc Degeneration.** With age, notochordal cells are gradually replaced by chondrocyte-like cells. Inflammation, ROS, and inadequate oxygen supply lead to cell cluster formation, cellular senescence, impaired autophagy, and cell death (apoptosis, necroptosis, pyroptosis, ferroptosis), releasing DAMPs triggering further inflammation. Endplate calcification lead to neovascularization, causing ROS accumulation and immune cell infiltration. Ultimately, the resulting imbalance between reduced anabolism and increased MMP and ADAMTS activity leads to ECM remodeling, proteoglycan loss, and altered collagen structure. Abbreviations: DAMPs, damage-associated molecular patterns; ROS, reactive oxygen species; MMPs, matrix metalloproteinases; ADAMTS, a disintegrin and metalloproteinase with thrombospondin motifs; ECM, extracellular matrix. Created in BioRender. https://BioRender.com/6t0e2od (URL accessed on 6 October 2025).

Collectively, the coordinated upregulation of MMPs, ADAMTS, and other proteases, unopposed by sufficient inhibitor activity, drives the ECM remodeling observed in IVDD, cleaving structural proteins such as collagens, aggrecan, and fibronectin into fragments that further disrupt the disc microenvironment. These extracellular changes, together with cellular processes such as inflammation, oxidative stress, and senescence, create a self-perpetuating degenerative network that accelerates disease progression. Figure 2 illustrates how these interconnected molecular and cellular mechanisms converge to drive the pathophysiology of IVDD.

## 6. Treatment Approaches for Intervertebral Disc Degeneration

Understanding and targeting the microenvironmental changes that drive and sustain degeneration is central to the development of effective treatments. Current approaches include conservative management, surgical interventions, and an expanding array of regenerative and molecular therapies aimed at restoring disc structure and function [139,140].

### 6.1. Conservative Management

Conservative (non-surgical) treatments remain the first-line approach for most patients with discogenic pain or mild-to-moderate IVDD. These include:

- Physical therapy and exercise: Targeted exercise regimens are designed to stabilize the spine, improve posture, and relieve pressure on affected discs. Mechanical loading within physiological limits can enhance nutrient diffusion and promote anabolic disc cell behavior [141,142].
- Pharmacologic agents: Non-steroidal anti-inflammatory drugs (NSAIDs), analgesics, and muscle relaxants are frequently used to manage pain and inflammation [139,143].
- Spinal injections: Epidural corticosteroid injections may provide temporary symptom relief by reducing perineural inflammation [144,145].

While these modalities focus on symptom management, they have minimal impact on halting or reversing disc degeneration.

### 6.2. Surgical Interventions

Surgical treatment is considered for patients with advanced degeneration, spinal instability, or neurological deficits refractory to conservative management. The most common procedures include:

- Discectomy: Removal of herniated or degenerated disc material that compresses nerve roots or the spinal cord. While effective for decompression, discectomy may accelerate adjacent segment degeneration due to biomechanical alterations [146,147].
- Spinal fusion: Interbody fusion is achieved using cage implants, usually supported with bone graft or other materials enhancing bone fusion. The stability of the motion segment is often further improved with the addition of transpedicular stabilization devices. Although fusion can relieve pain and improve stability, it eliminates motion at the treated level and may predispose adjacent segments to accelerated degeneration [148,149,150].
- Total disc replacement (TDR): Artificial disc implants aim to preserve segmental mobility while alleviating symptoms. Long-term data suggest favorable biomechanical outcomes, but device-related complications and patient selection remain critical considerations [151,152].

In recent years, there has been a growing emphasis on minimally invasive and endoscopic surgical techniques, which aim to reduce tissue trauma, shorten recovery time, and minimize postoperative complications while achieving comparable clinical outcomes. These approaches are increasingly used across various stages of disc degeneration, particularly in younger or more active patients [153,154].

Nonetheless, all of these structural interventions primarily address mechanical failure rather than regenerating native disc tissue or restoring physiological microenvironmental conditions.

### 6.3. Cell-Based Therapies

Mesenchymal stem cells (MSCs) are a promising regenerative approach for IVDD, owing to their ability to differentiate into nucleus pulposus-like cells and secrete bioactive factors that promote ECM synthesis, suppress inflammation, and support disc repair [155,156,157].

Preclinical studies have shown that MSC implantation restores disc height, improves hydration, and increases proteoglycan and collagen II content [158,159,160]. These effects are mediated by both differentiation and paracrine mechanisms, including suppression of IL-6, IL-8, and TNF-α [161,162]. In animal models, MSCs also improved pain without obvious radiological changes, suggesting functional benefits [163]. Clinical trials using autologous MSCs have reported pain relief, improved disc hydration, and functional gains [164,165,166,167]. A randomized study demonstrated superior outcomes in the group of five MSC-treated patients compared to controls [168], with long-term safety and efficacy confirmed in larger cohort of 24 patients [169].

However, challenges such as poor MSC survival in the harsh disc environment and unclear mechanisms of action persist [170]. Strategies like hypoxic preconditioning, biomaterial scaffolds, and MSC-derived exosomes are under investigation to enhance therapeutic efficacy [155].

While MSC therapy is advancing, interest has grown in notochordal cell-based therapies, inspired by the observation that NCs persist into adulthood in certain animal species resistant to disc degeneration [171,172]. Therapeutic strategies involving NCs include: 1. Direct transplantation of NCs or NC-rich tissue; 2. Use of NC-conditioned media or secretome, which enhances ECM production, inhibits inflammation, and supports NP cell proliferation; 3. Differentiation of pluripotent stem cells into NC-like cells, although standardization of phenotypic markers and confirmation of regenerative function remain areas for future study [172]. Evidence suggests that NCs promote ECM synthesis, prevent NP cell apoptosis, secrete anti-inflammatory and pro-regenerative factors such as TGF-β, TIMP-1, and GAG-rich ECM proteins, and act as chemoattractants, promoting the migration and differentiation of endogenous MSCs toward a regenerative NP phenotype [171]. Thus, NC-based therapy holds promise as a disease-modifying approach, potentially complementing or enhancing MSC-based strategies.

### 6.4. Growth Differentiation Factors

Growth factor (GF) therapy involves the injection of bioactive peptides into the IVD to stimulate ECM production, reduce catabolic activity, and suppress inflammation. GFs bind to cell surface receptors and activate signaling cascades that regulate protein synthesis, differentiation, apoptosis, and cell proliferation [173].

Among the most studied GFs in disc regeneration are growth differentiation factors (GDFs), especially GDF5 and GDF6, members of the TGF-β superfamily. In ovine and rabbit models, these factors have promoted anabolic ECM gene expression and inhibited inflammatory mediators such as IL-1β and TNF-α [174,175,176]. In particular, GDF6 induces robust NP-like differentiation of mesenchymal stem cells without triggering hypertrophy [160,177]. It also functions as a chemoattractant to chondrocytes and NP cells, and modulator of local inflammation [178,179].

In vivo studies have shown that GDF5 and GDF6 can enhance disc hydration, matrix integrity, and reduce pain-associated markers in animal models [174,175,176,180]. However, the short biological half-life of GFs, ranging from hours to days, poses a challenge for sustained therapeutic effects in chronic degeneration [181,182]. To overcome this, controlled-release delivery systems (e.g., microspheres, nanoparticles, viral vectors) have been developed to prolong bioactivity [183].

Other growth factors like TGF-β, BMPs (bone morphogenetic proteins), PDGF (platelet-derived growth factor), and EGF (epidermal growth factor) also exhibit anti-inflammatory and anti-catabolic properties by downregulating cytokines (IL-1, IL-6, TNF-α), MMPs, nitric oxide, and PGE2 [181,182]. A mice study revealed that TGF-β may also regulate NGF expression, linking it to pain modulation in IVDD [184]. Additionally, platelet-rich plasma (PRP) serves as a rich source of endogenous GFs, with PDGF shown to reduce apoptosis in AF cells under stress conditions [145,185].

### 6.5. Senolytics

Senolytics are a class of therapeutic agents designed to selectively eliminate senescent cells [186]. Senolytics like quercetin, curcumin, and o-vanillin have demonstrated efficacy in vitro and in vivo by reducing senescent NP cell burden, suppressing SASP expression, and alleviating ECM degradation [187,188,189,190]. Mice studies point that some senolytics, including dasatinib and fisetin, function via inhibition of the PI3K/Akt/mTOR pathway, while others such as ABT-263 (navitoclax) target BCL-2 family proteins to induce apoptosis selectively in senescent cells without affecting healthy ones [191,192,193].

In rat models, the delivery systems like nanoparticles have improved targeting of senolytics to the IVD, minimizing off-target effects and enhancing therapeutic precision [194]. Despite promising results in preclinical studies, clinical translation requires further investigation into the specificity, dosing, and long-term safety of senolytics in IVDD.

### 6.6. Ferroptosis Inhibition

Ferroptosis plays a key role in the pathogenesis of IVDD. Recent studies have demonstrated that inhibition of ferroptosis alleviates IVDD in cellular and animal models by preserving ECM homeostasis and reducing oxidative damage to disc cells [82].

Several strategies have emerged to suppress ferroptosis in the disc environment. In mice, iron chelators such as deferoxamine (DFO), deferiprone (DFP), and deferasirox (DFX) reduced intracellular iron accumulation, limiting the Fenton reaction and subsequent ROS production [195,196,197,198]. Antioxidants like ferrostatin-1 (Fer-1), liproxstatin-1 (Lip-1), and α-tocopherol inhibit lipid peroxidation and protect disc cells [199,200,201]. Upregulation of GPX4 or preservation of its activity via precursors (e.g., N-acetylcysteine) and cofactors (e.g., selenium) further reduces oxidative stress [202,203,204].

Regulatory pathways including the GPX4-GSH axis, FSP1-CoQ10 system, and NRF2 signaling are targets for ferroptosis inhibition. Additionally, modulation of lipid metabolism (via LOX or ACSL4 inhibition), iron export (via FPN upregulation), and mitochondrial ROS scavenging (e.g., mitoquinone) offer promising therapeutic angles [81,194,205].

Emerging evidence supports ferroptosis inhibitors as potential disease-modifying agents for IVDD, with several compounds demonstrating efficacy in vitro and in vivo. However, their translation into clinical practice requires further validation of safety, bioavailability, and disc-specific delivery systems.

### 6.7. Gene Therapy

Gene therapy represents a promising strategy to counteract IVDD by modifying gene expression to enhance ECM synthesis, suppress catabolism, and reduce inflammation. This approach has utilized both viral and non-viral vectors, as well as genome-editing tools [206].

Viral vector-mediated gene delivery has been successfully employed in preclinical models. Retroviral and adenoviral vectors have been used to introduce therapeutic genes like IL-1 receptor antagonist into NP cells, demonstrating sustained gene expression and anti-inflammatory effects [206,207,208,209]. Lentiviral delivery targeting MMP3 and Sox9 genes significantly attenuated IVDD and enhanced collagen II and aggrecan expression in rabbit lumbar discs [210].

Non-viral methods are also gaining traction due to safety concerns associated with viral vectors. Animal studies have demonstrated that RNA interference (RNAi) can effectively regulate inflammation by silencing pro-inflammatory genes such as toll-like receptor 4 (TLR4), resulting in protective effects in NP cells [211,212]. Ultrasound-targeted microbubble destruction (UTMD) has enhanced plasmid DNA delivery and gene expression duration in vivo [213]. Polyplex micelle-based delivery of miRNA-25-3p (Micro ribonucleic acid-25-3p) has reduced matrix degradation and supported ECM restoration in animal models [214]. Poly(ethylene glycol)-based polyplex micelles with hybrid shell architectures have shown particular promise as non-viral carriers in preclinical studies. By protecting nucleic acids from degradation, prolonging circulation, and enhancing cellular uptake, these ternary polyplex micelles (TPMs) achieve superior transfection efficiency and tissue accumulation compared with conventional systems. Incorporating such advanced carriers into gene therapy approaches for IVDD may improve therapeutic precision, bioavailability, and safety, thereby facilitating translation from preclinical studies to clinical application [215]. Building on these advances, thermo-responsive mixed polyplex micelles (MPMs) have emerged as another versatile platform. MPMs have been employed to transport heme oxygenase-1 (HO-1) plasmid DNA to NP cells, resulting in markedly higher transfection efficiency and stability than traditional micelles. In the same study, HO-1 overexpression reduced IL-1β-induced catabolic and inflammatory responses while enhancing ECM synthesis in vitro. In vivo administration attenuated inflammation and preserved disc structure in rats [216].

CRISPR/Cas9-based genome editing offers precise modification of key genes. Editing TNFR1 reduced MMP3 levels and increased aggrecan expression, while knockout of TRPV4 (Transient receptor potential cation channel subfamily V member 4) in human AF cells reduced inflammatory gene expression under mechanical stress in vitro [217,218]. Epigenome editing targeting AKAP150 (A-kinase anchoring protein 150) in dorsal root ganglia has been explored as a neuromodulatory therapy for IVDD-associated pain [219].

mTOR signaling, a central regulator of metabolism, has been linked to disc cell survival. Its inhibition via RNAi or rapamycin confers protection against apoptosis, senescence, and matrix breakdown in human NP cells in vitro [220]. Activation of the PI3K/Akt/mTOR axis may further enhance ECM synthesis and reduce catabolic activity [221,222].

Despite encouraging preclinical results, challenges such as off-target effects, immunogenicity, and vector safety must be addressed for successful clinical translation of gene therapies for IVDD [206].

### 6.8. Hydrogels and Scaffold-Based Therapies

Hydrogels have emerged as a promising biomaterial for IVD regeneration due to their biocompatibility, high water content, and mechanical properties that closely mimic the native NP tissue [223,224]. These three-dimensional hydrophilic polymer networks support IVD cell viability and matrix production while offering tunable biochemical and biomechanical properties, such as shear-thinning, ECM biomimicry, and injectability [225,226].

Various natural (e.g., alginate, chitosan, collagen) and synthetic (e.g., PEG, PVA) hydrogels have been tested, often in combination to form composite hydrogels that harness the biological activity of natural polymers and the structural integrity of synthetics [226,227]. Composite hydrogels have shown success in mimicking NP architecture, supporting stem cell differentiation, and modulating inflammatory responses [227,228,229]. Injectable hydrogels are particularly attractive due to their minimally invasive delivery and ability to conform to irregular defects [225,230].

In vitro and in vivo rat studies have demonstrated the potential of hydrogels as carriers for bioactive molecules (e.g., BMP-2, IL-4, miRNA) and therapeutic cells (e.g., MSCs), with promising outcomes in restoring disc height, promoting ECM synthesis, and reducing inflammation [231,232,233,234]. Advanced formulations, such as self-healing or gene-loaded hydrogels, further expand therapeutic possibilities by enhancing delivery, retention, and function in the harsh IVD microenvironment [235,236].

Recent advances include the development of conductive or “smart” scaffolds, which incorporate electromechanical signal transfer capabilities through conductive polymers or piezoelectric materials like aligned collagen fibers [183,237,238]. These may promote mechanosensitive cell responses and remodeling.

Despite these enhancements, challenges remain including degradation control, immunogenicity, and regulatory hurdles, which must be addressed to enable clinical translation. Nevertheless, hydrogels represent a compelling, multifunctional platform for biological repair of the degenerated IVD.

### 6.9. Nanomedicine Approaches

Nanomedicine offers promising strategies for addressing the challenges of IVDD, particularly by overcoming limitations in drug delivery caused by the avascular nature of the IVD [239]. Nanomaterials, due to their tunable properties, biocompatibility, and controlled drug release capabilities, can enhance therapeutic efficacy while minimizing systemic side effects [239,240].

Multiple nanocarrier systems have been explored, including nanoparticles, microspheres, gene-nanocomplexes, exosomes, and nanocomposite hydrogels. In mice, these platforms enabled passive, active, and physicochemical targeting, improving local drug retention and enabling delivery of anti-inflammatory agents, growth factors, and gene therapies [241,242,243,244].

Passive targeting systems, such as liposomal nanoparticles, exploit the disc’s inflammatory microenvironment to localize therapeutics, while active targeting systems, such as peptide-modified fullerenes, achieve cell-specific delivery [240,243]. Physicochemical strategies, including ultrasound-triggered release, allowed externally controlled targeting and enhanced penetration of IVD tissue in in vitro and animal studies [244,245,246].

Nanoparticles have been used to promote anabolic metabolism, suppress inflammation and fibrosis, and stimulate stem cell migration and differentiation. For instance, dual-growth-factor-loaded nanoscaffolds enhanced MSC differentiation and ECM synthesis [247]. Gene-nanoparticle complexes enable non-viral delivery of anti-inflammatory and anti-fibrotic genes, with agents like HO-1 and NR4A1 (nuclear receptor 4A1) showing therapeutic efficacy in bovine models [216,248].

Biologically derived nanocarriers, such as exosomes from MSCs and NPCs, deliver miRNAs or proteins that reduce apoptosis, oxidative stress, and inflammation, while also promoting regeneration. Nanocomposite hydrogels reinforce mechanical properties of the disc and support cell viability, offering injectable platforms for tissue repair [249].

Overall, nanomedicine introduces a versatile and targeted approach to IVDD treatment by addressing its complex biochemical, cellular, and mechanical pathologies. However, clinical translation requires further investigation into delivery efficiency, long-term safety, and scalable manufacturing.

**Figure 3:**
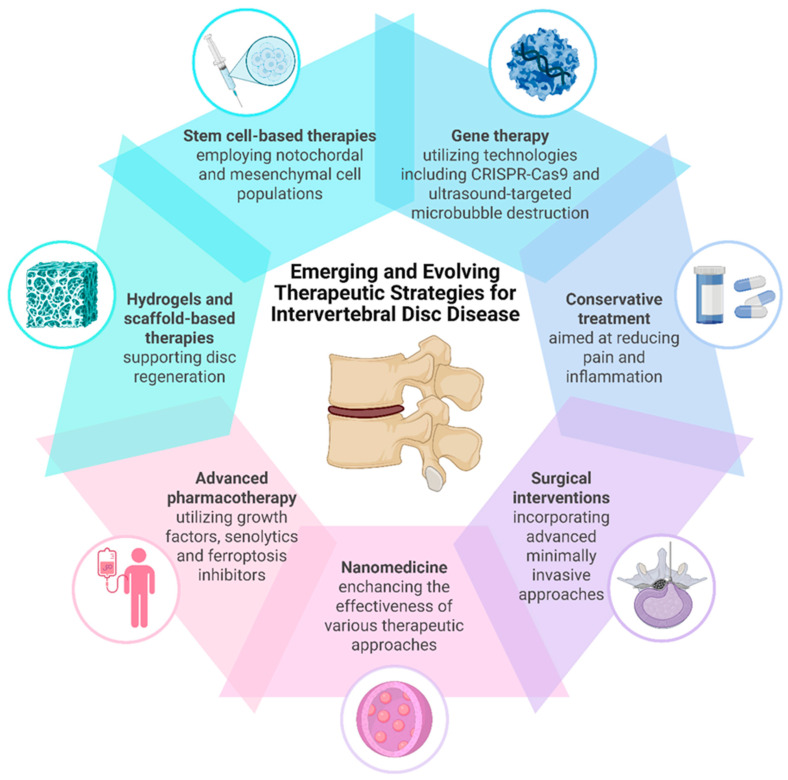
**Emerging and Evolving Therapeutic Strategies for Intervertebral Disc Disease.** A range of innovative therapeutic approaches is currently being explored to address the multifactorial nature of intervertebral disc disease. Stem cell-based therapies, employing notochordal and mesenchymal stem cell populations, aim to restore disc homeostasis and promote regeneration. Gene therapy strategies, including CRISPR-Cas9 and ultrasound-targeted microbubble destruction, offer the potential for precise modulation of pathogenic molecular pathways. Hydrogels and scaffold-based therapies provide structural support and biochemical cues to facilitate disc tissue regeneration. Advanced pharmacotherapy targets key molecular drivers of degeneration using growth factors, senolytics, and ferroptosis inhibitors. Nanomedicine is being developed to enhance delivery efficiency and therapeutic precision across multiple intervention types. Surgical interventions, particularly minimally invasive techniques, continue to evolve to reduce procedural morbidity while addressing structural degeneration. Conservative treatments such as pharmacologic pain management and physical therapy remain integral for symptomatic relief and functional maintenance. Created in BioRender. https://BioRender.com/g9vwerv (URL accessed on 6 October 2025).

Collectively, these strategies reflect a translational shift from symptomatic treatment toward biologically informed, disease-modifying therapies for intervertebral disc degeneration. Figure 3 illustrates the spectrum of therapeutic modalities for intervertebral disc disease.

## 7. Discussion

The review highlights the intervertebral disc microenvironment as a dynamic and multifactorial niche where structural, biochemical, and cellular perturbations converge to drive intervertebral disc degeneration. Degeneration is not the result of a single pathological trigger but of a complex, self-perpetuating cascade. A critical implication is that targeting a single pathway may provide incomplete benefit unless the broader network of feedback loops is considered.

Alterations in extracellular matrix composition reduce the disc’s ability to retain water and maintain normal mechanical properties, leading to loss of disc height and increased tissue stiffness. These biomechanical changes alter the distribution of mechanical loads within the disc, increasing stress on the annulus fibrosus and cartilage endplates. Over time, this abnormal load transmission accelerates microdamage to disc structures, disrupts cellular mechanotransduction pathways, and stimulates the production of catabolic enzymes and pro-inflammatory mediators. As a result, the structural and biochemical integrity of the disc deteriorates further, reinforcing the degenerative cascade.

Simultaneously, endplate calcification decreases the permeability of the cartilage endplates, impairing the diffusion of essential nutrients and oxygen into the nucleus pulposus while limiting the removal of metabolic waste. The nutrient-deprived, acidic, and pro-oxidative microenvironment, as well as the ingrowth of blood vessels permitted by structural disruption of the annulus fibrosus, facilitate immune cell infiltration into normally sequestered regions and promote sustained inflammatory activation, thereby accelerating the degenerative cascade.

The concept of the disc as an immune-privileged organ is central to understanding IVDD pathogenesis. Once structural integrity is breached, infiltration of macrophages and other immune cells fuels chronic inflammation and fibrosis. The dynamic balance between M1 and M2 macrophage phenotypes illustrates both the potential for repair and the risk of maladaptive remodeling.

In parallel, cellular senescence emerges as a critical mediator linking inflammation, oxidative stress, and matrix degradation. Senescent disc cells not only lose their ability to maintain tissue homeostasis but also acquire a senescence-associated secretory phenotype, characterized by sustained production of pro-inflammatory cytokines, chemokines, and matrix-degrading enzymes. This secretory activity reinforces macrophage recruitment and polarization toward pro-inflammatory phenotypes, amplifies oxidative stress, and drives further extracellular matrix breakdown, thereby integrating cellular aging into the broader degenerative network.

The degenerative cascade is amplified by the interplay of cell death pathways—including apoptosis, pyroptosis, necroptosis, and ferroptosis—each contributing to cell loss and inflammatory amplification. These processes are not mutually exclusive but interwoven, with shared upstream regulators such as TNF-α, IL-1β, NF-κB, and MAPK coordinating catabolic and inflammatory signaling. The inflammatory cytokines such as TNF-α and IL-1β not only drive ECM degradation but also induce oxidative stress, which in turn promotes mitochondrial dysfunction and further activates multiple cell death pathways. Autophagy impairment exacerbates these effects by reducing cellular stress tolerance and allowing damaged organelles to accumulate, thereby intensifying both oxidative and inflammatory signaling.

Many preclinical studies still assess IVDD microenvironment in static terms, rather than capturing the temporal and spatial heterogeneity of cell phenotypes and ECM structure during disease progression. Improved in vivo models and single-cell approaches are needed to map the numerous dynamic interactions more precisely.

For decades, the management of IVDD has relied primarily on symptom-oriented approaches, including conservative treatments such as physical therapy, analgesics, and rehabilitation, as well as surgical interventions such as discectomy and spinal fusion in advanced cases. While these strategies remain the current clinical standard, they primarily address pain and functional impairment without modifying the underlying degenerative processes. Moreover, surgical procedures may alter spinal biomechanics, potentially accelerating degeneration in adjacent segments.

In recent years, there has been a growing emphasis on the development of biological and regenerative therapies that aim to intervene at the molecular and cellular levels. Emerging therapeutic strategies, including mesenchymal and notochordal cell transplantation, senolytics, ferroptosis inhibitors, gene therapy, and nanomedicine-based delivery systems, reflect a paradigm shift toward disease-modifying interventions. Yet, despite compelling preclinical results, their clinical translation remains constrained by challenges in safety, reproducibility, and long-term efficacy. Cell-based therapies illustrate both promise and limitation. While mesenchymal stem cells improve disc hydration and function in early-phase trials, their survival in the harsh disc microenvironment is poor, and mechanisms of action remain incompletely understood. Notochordal cell-derived approaches are conceptually attractive given their persistence in non-degenerative species, but the lack of standardized phenotypic markers hinders progress. Similarly, senolytics and ferroptosis inhibitors directly target molecular hallmarks of degeneration, but questions remain about disc-specific delivery, off-target effects, and durability of response.

Gene and biomaterial-based strategies add further complexity. Hydrogels and scaffolds mimic the disc niche and provide platforms for controlled therapeutic delivery, but variability in degradation kinetics, immunogenicity, and mechanical properties remains problematic. Nanocarrier systems enhance precision but face regulatory and manufacturing hurdles before large-scale application. Collectively, these approaches emphasize the need for combinatorial or staged therapies that address the multifactorial nature of IVDD, rather than isolated interventions.

Despite substantial preclinical advances, there is a notable translational gap. Current animal models, while valuable, do not fully recapitulate the human disc’s anatomy, biomechanics, or slow degenerative course. Clinical studies are often limited by small cohorts, heterogeneous patient populations, and short follow-up durations. Furthermore, most outcome measures focus on pain and function, with limited correlation to molecular or structural biomarkers of disc health. This gap underscores the need for standardized clinical endpoints and the integration of imaging, biochemical, and biomechanical metrics to better assess therapeutic efficacy. Another challenge is patient stratification. IVDD represents a heterogeneous spectrum influenced by genetic predisposition, systemic metabolic status, mechanical loading, and lifestyle factors. Future trials should prioritize personalized approaches, potentially incorporating genomic, proteomic, and imaging biomarkers to identify patients most likely to benefit from specific interventions.

## 8. Conclusions

Intervertebral disc degeneration is a multifactorial process involving an intricate interplay of biochemical, cellular, and mechanical alterations within the disc microenvironment. The transition from a homeostatic to a degenerative niche is driven by extracellular matrix degradation, inflammatory signaling, oxidative stress, aberrant angiogenesis, and dysregulated cell death mechanisms including apoptosis, pyroptosis, and ferroptosis. These processes collectively impair disc structure and function, contributing to chronic pain and disability.

Understanding the pathological transformations within the disc microenvironment has catalyzed the development of a new generation of targeted therapies. These include cell-based approaches such as mesenchymal stem cells and notochordal cell derivatives, senolytics to clear dysfunctional cells, ferroptosis inhibitors to mitigate oxidative injury, and gene therapy strategies aimed at restoring anabolic–catabolic balance. Concurrently, biomaterial innovations, including hydrogels, scaffolds, and nanocarrier systems, are enhancing the precision and durability of therapeutic delivery.

Despite encouraging advances in preclinical and early clinical studies, challenges remain in translating these therapies into widespread clinical practice. Future research must prioritize the integration of multi-modal interventions, long-term safety evaluation, and the development of patient-specific treatment algorithms. Ultimately, an improved understanding of the disc microenvironment holds the promise of shifting IVDD management from symptomatic relief to true disease modification.

## 9. Future Directions

Taken together, the presented insights suggest that progress in IVDD management will depend on an integrated strategy combining deeper mechanistic understanding of disc microenvironmental dynamics, development of multimodal therapies targeting multiple degenerative pathways, and rigorous translational frameworks bridging bench to bedside. Systems biology and computational modeling may provide powerful tools to unravel the interdependencies among catabolic signaling, immune responses, and mechanical stress, thereby informing rational combination therapies. Importantly, the field must also address regulatory, ethical, and economic considerations to enable broad clinical translation of emerging interventions.

## Figures and Tables

**Table 1:** Summary of the key cytokines and chemokines involved in the inflammatory processes of intervertebral disc degeneration. IVD—Intervertebral disc, IVDD—Intervertebral disc degeneration, NLRP3—NOD-like receptor protein 3, MMPs—Matrix metalloproteinases, ADAMTs—A disintegrin and metalloprotease with thrombospondin motifs, IL—Interleukin, TNF-α—Tumor necrosis factor alpha, IFN-γ—Interferon-γ, TGF-β—Transforming growth factor-beta, CCL—Chemokine ligand, PGE2—Prostaglandin E2, NK—Natural killer, MAPK—Mitogen-activated protein kinase, NF-κB—Nuclear factor kappa-light-chain-enhancer of activated B cells, C/EBP—CCAAT-enhancer-binding protein, β-NGF—β-nerve growth factor.

Cytokine/Chemokine	Source (Cells)	Expression in IVDD	Primary Role in IVDD	References
IL-1β	- IVD cells - Immune cells (macrophages, monocytes, dendritic cells, B cells, natural killer cells)	- Higher expression in degenerated and painful discs - Increased by NLRP3 activation	- Master pro-inflammatory cytokine - Drives matrix degradation, angiogenesis, cell death, senescence - Central upstream regulator of inflammatory and catabolic cascades - Upregulates: IL-6, IL-8, IL-17, TNF-α, CCL3, MMP-1, MMP-3, MMP-9, MMP-10, MMP-13, ADAMTS-4 - Downregulates: collagen type II	[94,95,96,98,99,100,101,103,106]
TNF-α	- IVD cells - Immune cells (macrophages, monocytes, dendritic cells, B cells, natural killer cells, type 1 T-helper cells)	- Higher expression in symptomatic compared to asymptomatic discs	- Induces catabolism, autophagy, apoptosis, senescence, vascularization - Leads to the sensitization of nerve roots, which increases pain perception - Acts synergistically with IL-1β - Upregulates: IL-6, IL-8, IL-17, CCL3, MMP-1, MMP-3, MMP-13, ADAMTS-4, ADAMTS-5 - Downregulates: collagen type II	[94,95,96,97,98,101,103,105,106]
IL-6	- IVD cells - Immune cells (T cells, type 2 T-helper cells, macrophages)	- Higher expression in degenerated discs - Induced by IL-1β and TNF-α - Higher expression in painful discs	- Amplifies IL-1 and TNF actions - Links inflammation with neuropathic pain by inducing apoptosis of neuronal cells in dorsal root ganglion - Upregulates: PGE2, MMP-2, MMP-13 - Downregulates: collagen type II	[95,96,98,101,102,103,104,106]
IL-8	- IVD cells	- Higher expression in degenerated discs - Induced by IL-1β and TNF-α	- Induces hyperalgesia by stimulating local sympathetic amine release	[95,98,101,103,104]
IL-17	- IVD cells - Immune cells (type 17 T-helper cells, other lymphocytes, neutrophils, mast cells)	- Higher expression in degenerated discs - Induced by IL-1β and TNF-α	- Stimulates chemokines; recruits immune cells; amplifies inflammation - Potentiates IL-1 and TNF effects	[95,96,98,105]
IFN-γ	- Immune cells (type 1 T-helper cells, cytotoxic T cells, natural killer cells, macrophages, myeloid cells, dendritic cells)	- Higher expression in degenerated discs	- Enhances inflammatory mediator release - Linked to herniation and immune activation	[96,104]
IL-4	- Type 2 T-helper cells - Macrophages, monocytes	- Higher expression in degenerated discs	- Anti-inflammatory, analgesic; inhibits pro-inflammatory cytokines - Opposes IL-1 and TNF signaling	[96,103]
CCL-3	- IVD cells	- Higher expression in degenerated discs - Induced by IL-1β and TNF-α	- Chemotactic and pro-inflammatory; activates MAPK, NF-κB, and C/EBP signaling pathways - Recruits immune cells to degenerative sites	[95,98,104]
CCL-5	- IVD cells	- Higher expression in degenerated and painful discs - Induced by IL-1β and TNF-α	- Mediates inflammatory response in disc herniation - Linked to inflammation-associated pain	[95,96,98,99,103]
β-NGF	- Endothelial cells lining the IVD neovasculature	- Increased in painful discs	- Promotes nerve ingrowth which is associated with discogenic pain	[96,104]

**Table 2:** Key matrix metalloproteinases involved in intervertebral disc degeneration. MMP—matrix metalloproteinase, IVD—intervertebral disc, IVDD—intervertebral disc degeneration, ECM—extracellular matrix, proMMP—promatrix metalloprotease, AF—annulus fibrosus.

MMP	Major Matrix Substrates	Primary Role in IVDD	Key Features	References
MMP-1 (Collagenase-1)	Intact interstitial collagen (type I-III, VII, X), aggrecan	Initiates collagen degradation	- Degrades the intact collagen - Activates MMP-9 - Marker of initial degeneration	[122,123,124,125,126]
MMP-2 (Gelatinase A)	Denatured collagen of the basement membrane (collagen type IV-VI, X), gelatin, elastin, fibronectin	Degrades denatured collagens	- Involved in matrix remodeling - Remodels denaturated basement membranes	[123,124,126]
MMP-3 (Stromelysin-1)	Collagen (type II, IV, IX), proteoglycans, elastin, fibronectin, aggrecan, laminin	Broad ECM degradation and MMP activation	- Activates other MMPs, which amplifies matrix degradation - Broad substrate specificity, driving extensive ECM breakdown	[122,123,126,127]
MMP-7 (Matrilysin)	Gelatin (type I, II, IV, V), fibronectin, proteoglycans, aggrecan, collagen (type II, IV–X)	Extensive matrix remodeling	- Targets a broad range of extracellular matrix components - Activates proMMP (-1, -2, -9)	[126,129]
MMP-8 (Neutrophil collagenase)	Collagen (type I–III)	Collagen degradation	- Breaks down the helical region of the fibrillar collagens	[127]
MMP-9 (Gelatinase B)	Gelatin, denatured collagen (type IV, V, VII, X, XIV), aggrecan, elastin, fibrillin, osteonectin	Basement membrane and ECM degradation	- Degrades various structural elements of the extracellular matrix - Negatively correlates with pain	[126]
MMP-10	Collagen (type III-V), gelatin, aggrecan, elastin	ECM degradation	- Degrades non-collagenous matrix proteins and denatured collagens - Activates proMMP (−1, −8, −11, −13)	[126]
MMP-12 (Metalloelastase)	Aggrecan, elastin, collagen (type I, IV), gelatin, fibronectin, laminin, vitronectin, proteoglycan	ECM degradation	- Targets a broad range of extracellular matrix components - Associated with myofibroblast activity	[126]
MMP-13 (Collagenase-3)	Fibrillar collagens (esp. type II, also I and III)	Late-stage ECM degradation	- Promotes disc calcification and fibrosis	[121,122,125,126]
MMP-14 (MT1-MMP)	Collagen (type I-III), gelatin, fibronectin, laminin, aggrecan	Cell-surface protease activator	- Regulates expression of MMP-2 on AF cell surface	[124,126]
MMP-19	Basement membrane collagen type IV, gelatin type I, aggrecan, fibronectin, laminin	Inhibits angiogenesis	- Restrains angiogenesis and prevents vascular ingrowth in degenerative IVD tissue	[126]

## Data Availability

No new data were created or analyzed in this study. Data sharing is not applicable to this article.

## Abbreviations

The following abbreviations are used in this manuscript:

AAV	Adeno-associated viruses
ACSL4	Acyl-CoA synthetase long chain family member 4
ADAMTS	A disintegrin and metalloproteinase with thrombospondin motifs
AF	Annulus fibrosus
AGE	Advanced glycation end-product
AIM2	Absent in melanoma 2
AKAP150	A-kinase anchoring protein 150
AMPK	AMP-activated protein kinase
ANGPTL4	Angiopoietin-like 4
ASC	Apoptosis-associated speck-like protein containing a CARD
Atg	Autophagy-related protein
BDNF	Brain-derived neurotrophic factor
BMP	Bone morphogenetic protein
BMP-7	Bone morphogenetic protein-7
β-NGF	β-nerve growth factor
CA HIF-1α	Constitutively active form of HIF-1α
CCL	Chemokine ligand
CEP	Cartilage endplates
C/EBP	CCAAT-enhancer-binding protein
CGRP	Calcitonin gene-related peptide
COX-2	Cyclooxygenase-2
DAMPs	Damage-associated molecular patterns
DFO	Deferoxamine
DFP	Deferiprone
DFX	Deferasirox
ECM	Extracellular matrix
EGF	Epidermal growth factor
ER	Endoplasmic reticulum
ESCRT	Endosomal sorting complex required for transport
FAK	Focal adhesion kinase
FasL	Fas ligand
Fe^2+^	Ferrous iron
Fer-1	Ferrostatin-1
FPN/SLC40A1	Ferroportin
GDF	Growth differentiation factor
GF	Growth factor
Glut-1	Glucose transporter 1
Glut-3	Glucose transporter 3
GM-CSF	Granulocyte-macrophage colony stimulating factor
GPX4	Glutathione peroxidase 4
GSDMD	Gasdermin D
GSDMD-NT	N-terminal pore-forming GSDMD fragment
GSDME	Gasdermin E
GSH	Glutathione
HIF-1α	Hypoxia-inducible factor-1 alpha
HIF-2α	Hypoxia inducible factor 2α
HO-1	Heme oxygenase 1
HTRA1	High-temperature requirement protein A1
IFN-γ	Interferon-γ
IGF-1	Insulin-like growth factor-1
IL	Interleukin
iNOS	Inducible nitric oxide synthase
IRF1	Interferon regulatory factor 1
IVD	Intervertebral disc
IVDD	Intervertebral disc degeneration
LC3-II	LC3-phosphatidylethanolamine conjugate
Lip-1	Liproxstatin-1
LOX	Lipoxygenase
LPCAT3	Lysophosphatidylcholine acyltransferase 3
MAPK	Mitogen-activated protein kinase
miRNA	Micro ribonucleic acid
MLKL	Mixed lineage kinase domain-like protein
MMP	Matrix metalloproteinase
MSC	Mesenchymal stem cell
mTOR	Mammalian/Mechanistic target of rapamycin
mTORC1	Mammalian target of rapamycin complex 1
NC	Notochordal cell
NF-κB	Nuclear factor kappa-light-chain-enhancer of activated B cells
NGF	Nerve growth factor
NK	Natural killer
NLRC4	NLR family CARD domain-containing protein 4
NLRP3	NLR family pyrin domain containing 3
NO	Nitric oxide
NP	Nucleus pulposus
NR4A1	Nuclear receptor 4A1
NSAID	Non-steroidal anti-inflammatory drug
OPN	Osteopontin
PAMP	Pathogen-associated molecular pattern
PDGF	Platelet-derived growth factor
PGE2	Prostaglandin E2
PRP	Platelet-rich plasma
PUFA	Polyunsaturated fatty acid
Rb	Retinoblastoma protein
RIPK1	Receptor-interacting serine/threonine-protein kinase 1
RIPK3	Receptor-interacting serine/threonine-protein kinase 3
RNAi	RNA interference
ROS	Reactive oxygen species
SASP	Senescence-associated secretory phenotype
SA-β-gal	Senescence-associated β-galactosidase
SIPS	Stress-induced premature senescence
SLRP	Small leucine-rich proteoglycan
SOD	Superoxide dismutase
STAT3	Signal transducer and activator of transcription 3
sTNF-α	Soluble tumor necrosis factor-alpha
TACE	Tumor necrosis factor-alpha converting enzyme
TAZ	Transcriptional co-activator with PDZ-binding motif
TDR	Total disc replacement
TFR1	Transferrin receptor
TGF-β	Transforming growth factor-beta
TIMP	Tissue inhibitor of metalloproteinase
TLR4	Toll-like receptor 4
tmTNF-α	Transmembrane tumor necrosis factor-alpha
TNFR1	Tumor necrosis factor receptor 1
TNFR2	Tumor necrosis factor receptor 2
TNF-α	Tumor necrosis factor-alpha
TRPV4	Transient receptor potential cation channel subfamily V member 4
ULK1	Unc-51-like kinase 1
UTMD	Ultrasound-targeted microbubble destruction
VEGF	Vascular endothelial growth factor
YAP	Yes-associated protein
